# Pregnancy-Associated Alterations of Peripheral Blood Immune Cell Numbers in Domestic Sows Are Modified by Social Rank

**DOI:** 10.3390/ani9030112

**Published:** 2019-03-23

**Authors:** Christiane Schalk, Birgit Pfaffinger, Sonja Schmucker, Ulrike Weiler, Volker Stefanski

**Affiliations:** Department of Behavioral Physiology of Livestock, Institute of Animal Science, University of Hohenheim, Garbenstr 17, 70599 Stuttgart, Germany; christiane_schalk@uni-hohenheim.de (C.S.); birgit.pfaffinger@uni-hohenheim.de (B.P.); weiler@uni-hohenheim.de (U.W.)

**Keywords:** blood leukocyte subsets, cortisol, gestation, pig, reproduction, social rank

## Abstract

**Simple Summary:**

During pregnancy, the maternal immune system is characterized by changes in various immune functions. Nevertheless, pregnancy-associated immune changes and the interplay of rank-associated and gestation-induced immunomodulations are still poorly investigated in sows. Important aspects of blood cellular immunity, cortisol concentrations, and the influence of social rank position during pregnancy were investigated. The results showed that the numbers of various lymphocyte subpopulations decreased, while neutrophils and plasma cortisol concentrations increased during pregnancy. Those pregnancy-associated alterations in the immune system were affected especially in middle-ranking sows, indicating that social rank can influence the immune system and endocrine status in sows during pregnancy.

**Abstract:**

During pregnancy, the maternal immune system is characterized by a shift from adaptive to innate immune functions. Besides, the immune system can be influenced by social rank. Detailed knowledge of pregnancy-associated immune changes and of the interplay of rank-associated and gestation-induced immunomodulations is still fragmentary in sows. This study investigates both the numbers of various blood leukocyte subpopulations during pregnancy and the influence of social rank position on progressing pregnancy-associated alterations in group-housed sows. Sows were classified as low (LR), middle (MR), or high-ranking (HR). Five blood samples were collected from each of the 35 sows throughout pregnancy to evaluate the distribution of blood lymphocyte subpopulations and plasma cortisol concentrations. The numbers of T, natural killer (NK), and B cells, cytotoxic T cells (CTL), and CD8^+^ γδ- T cells decreased during the last trimester of pregnancy, while neutrophils and plasma cortisol concentration increased before parturition. Social rank revealed different effects on B cells and monocytes with MR sows showing higher numbers than LR sows. Plasma cortisol concentrations also tended to be higher in MR sows as compared to LR sows. In conclusion, sows show pregnancy-associated alterations in the immune system, which are influenced by social rank, as middle-ranking sows in particular display signs of stress-induced immunomodulations.

## 1. Introduction

The maternal immune system undergoes unique changes during gestation. Profound alterations in numbers and functionality of immune cells occur from the first trimester of pregnancy onwards. These adjustments are generally characterized by a shift from adaptive to innate immune functions [1,2]. On the level of the peripheral blood, this change is reflected in increased numbers of circulating granulocytes, monocytes, and dendritic cells (DC) while the numbers of T, B, and natural killer (NK) cells decrease [3,4,5,6,7]. This immune status, unique to pregnancy, is crucial for reproductive performance by achieving a successful pregnancy, but also for maternal health [8,9,10]. In contrast to rodents and humans, knowledge on pregnancy-associated immune changes in sows is fragmentary. Previous research investigated either broad measures of leukocyte blood counts [11,12,13,14] or an analysis of functionally distinct lymphocyte subsets, such as T helper (TH) cells, cytotoxic T cells (CTL), B cells, and NK cells, was not conducted during the entire pregnancy [11,15,16].

Moreover, it is well established that the social environment has a profound impact on the immune system in group-living species, including the pig [16,17,18,19,20], and that the immune competence of an individual can be strongly related to its social rank [4,21]. However, little is known on the interplay of rank-associated and gestation-induced immunomodulations in sows. This is rather surprising, considering that group-housing of sows is increasing worldwide and is mandatory, e.g., in the European Union (EU directive 2001/88/EC), for most of the pregnancy. In sows, several behavioral studies indicate that a low or intermediate social rank is associated with received aggressive behavior, poorer physical condition, lower body weight gain [22,23,24], and higher cortisol concentrations [23,25,26,27,28]. Considering the well-known immunomodulatory properties of cortisol [29,30], an effect of social rank on the T and B cell distribution is likely, with possible consequences for maternal and fetal health as well. However, most of the studies to date found no effects of social status on immune measures, such as the numbers of blood granulocytes and lymphocytes, lymphocyte proliferation, or antibody response, in pregnant sows [15,27,31]. Only Pacheco and Salak-Johnson [32] reported higher plasma cytokine levels (Interleukin-12) in blood plasma, and a reduced percentage of blood neutrophils in submissive pregnant sows as compared to dominants. Nevertheless, the conclusion that there is only little interplay between social status and pregnancy-associated immune system alterations should be considered premature as long as an assessment of the distribution pattern of the distinct lymphocyte subsets is missing. This view is also supported by data from non-pregnant female pigs, in which the clear influence of social rank on blood immune cell numbers after regrouping or changes in thermal environment is evident [33,34].

The aim of the present study was to investigate the numbers of various blood leukocyte subpopulations during all trimesters of pregnancy in group-housed multiparous sows with special emphasis on the question whether the social status of sows affects the progress of pregnancy-associated alterations.

## 2. Materials and Methods

### 2.1. Animals and Housing

A total of 40 multiparous German Landrace sows (parity number 4.3 ± 1.8) in two replicates of 20 sows each was included in this study. All animals were kept at the Agricultural Experiment Station of the University of Hohenheim (Unterer Lindenhof, Eningen unter Achalm, Germany) and housed according to the ethical and animal care guidelines (approved by the Committee on the Ethics of Animal Experiments of the Regional Commission of Tübingen, Germany; Ethical approval code: HOH 29/13). All sows were artificially inseminated with Pietrain semen and housed in groups of five animals, which were balanced by parity, body mass, and age at the beginning of the experiment. The sows were 819.4 ± 319.5 days of age and weighed 229.9 ± 41.4 kg at the beginning of pregnancy.

During gestation, sows had free access to water and were fed once a day at 08:00 AM with a standard barely-wheat-oat-based diet according to the actual metabolic requirements for pregnant sows (≤day 84 of pregnancy: 2.5 kg/day; ≥day 85 of pregnancy: 3.0 kg/day). Pens (approximately 16 m^2^) consisted of concrete flooring and solid wooden walls that restricted physical and visual contact between groups. For individual identification, all sows were marked with a commercial colored spray on the back and both sides of the body. All animals remained in a constant group composition over a period of four weeks after insemination. Afterwards, sows were housed in stable or dynamic group compositions (4 groups; dynamic from week 11–4 pre partum). In the dynamic groups, the composition was changed twice a week over eight weeks until four weeks before farrowing while the other sows stayed in a stable group composition during the entire pregnancy.

### 2.2. Blood Collection

Five blood samples were collected via jugular vein puncture during gestation from each pregnant sow (individual sampling duration <2 min). For blood sampling, sows were restrained using a nose snare. Blood samples were collected between 09:00–10:00 AM into lithium heparin tubes and K3 EDTA tubes (both Sarstedt, Nümbrecht, Germany). One sample was obtained during the first trimester of pregnancy (week 12 pre partum), two additional samples during the second trimester (week 10 and 7 pre partum), and two samples during the last trimester of pregnancy (week 4 and 2 pre partum). All samples were processed within 3 h after sampling.

### 2.3. Cortisol Measurement

Cortisol concentrations were determined radioimmunologically in blood plasma samples after ethanolic extraction as described previously [35]. Plasma was obtained from heparinized blood samples by centrifugation (1000× *g* for 10 min at 10 °C) and stored at −20 °C until assayed. [1.2,-3H]-cortisol (50 Ci/mmol, Hartmann Analytic, Braunschweig, Germany) was used as a tracer. Standards were prepared in charcoal-stripped plasma with final concentrations ranging from 2 to 200 ng cortisol/mL (Sigma-Aldrich, Munich, Germany). The coefficients of the inter-assay variation ranged between 13.9% and 8.5% depending on the endogenous concentration of the samples (13.6 and 39.9 ng/mL, respectively). The coefficient of intra-assay variability was 8.4%. The precision was determined by calculating the recovery rate of cortisol spiked samples (10, 20, and 40 ng/mL) and was 91% on average.

### 2.4. Differential Blood Count

The percentage of leukocyte and lymphocyte subpopulations in heparinized whole-blood was quantified by flow cytometry by a three-color immunofluorescent antibody staining procedure as described previously [36]. In detail, specific immune cell populations were characterized by use of different combinations of the following fluorochrome-labeled pig-specific antibodies directed against the cell surface markers: CD3 (clone PPT3), CD4 (clone 74-12-4), CD8α (clone 76-2-11), and CD172a (clone 74-22-15) (all SouthernBiotech, Birmingham, AL, USA). In brief, peripheral blood mononuclear cells (PBMC) and granulocytes were identified by their forward and side scatter characteristics. Granulocyte subpopulations were further differentiated into neutrophils and eosinophils by autofluorescence characteristics within the unstained control sample. PBMC subpopulations were classified by light scatter properties and the combination of surface marker expression as follows: T cells (SSC^−^ CD3^+^), naive T helper (TH) cells (SSC^−^ CD3^+^ CD4^+^ CD8α^−^), antigen-experienced TH cells (SSC^−^ CD3^+^ CD4^+^ CD8α^+^), cytotoxic T cells (CTL) (SSC^−^ CD3^+^ CD4^−^ CD8α^high^), γδ-T cells (SSC^−^ CD3^+^ CD4^−^ CD8α^−/dim^), natural killer (NK) cells (SSC^−^ CD3^−^ CD8α^+^), and monocytes (SSC^−^ CD3^−^ CD8α^−^ CD172a^high^). B cells were characterized by negative discrimination (SSC^−^ CD3^−^ CD8α^−^ CD172a^−^), which should characterize no other porcine blood immune cell fraction than B cells.

Total leukocyte counts were determined in EDTA whole blood by an automated hematology system (pocH 100-iV Diff, Sysmex, Norderstedt, Germany) and absolute numbers of the different immune cell populations in the blood were determined by combining cell frequencies obtained from the flow cytometric assessment with these total leukocyte counts.

### 2.5. Behavioral Recording and Rank Classification

Behavior patterns and their descriptions are listed in the ethogram shown in Table 1 [37,38].

Agonistic behavior was analyzed with video cameras (Viewex-350/WS; Monacor International, Bremen, Germany) by continuous recording for four hours at defined time-points during the entire pregnancy (week 13, 10, 8, 6, 4, and 2 pre partum) to determine the number of agonistic interactions, the initiating sow, and the outcome (win or defeat).

The data gathered from the recorded interactions per group were transferred into a winner-loser matrix. Based on these matrices, a modified Average Dominance Index (ADI) for each sow was calculated by using DomiCalc [39] following the formula:

$$\mathrm{ADI}=\frac{1}{n}\Sigma \omega_{ij}$$

Per pair of individuals, ω*_ij_* is calculated as the number of times a sow, *i*, won against another sow, *j (x_ij_)*, divided by the total number of agonistic interactions between the two sows, thus *ω_ij_ = x_ij_/(x_ij_ + x_ji_).* The ADI value of one individual is the average of all its dominance indices with all its interaction partners and varies from 0 to 1, with a higher value indicating a higher dominance rank in the group [40,41].

For each sow, an average ADI was calculated (mean value resulting from six observations). Animals receiving a mean value between 0.00–0.33 were categorized as low-ranking (LR), between 0.34–0.65 as middle-ranking (MR), and between 0.66–1.00 as high-ranking (HR) sows.

### 2.6. Statistical Analyses

Data of 35 pregnant sows (HR: 8; MR: 15; LR: 12) were included in the statistical analyses as sows with recurring estrous were excluded and one sow had to be removed from the experiment due to leg injuries.

Statistical models used R programming language version 3.1.0 [42] and the function “lmer” of the R package lme4 [43]. Linear mixed-effect models included the factors week of pregnancy, social rank, and their interaction as fixed effects as well as housing, replicate, and individual as random effects. Sampling duration and age were included as covariates in each model and were excluded if no significant effects (*p* > 0.1) could be observed. Body mass and number of litters were not included due to a positive correlation with age.

Residuals of immunological parameters and cortisol were examined for normal distribution with the Shapiro-Wilk test, as well as for homogeneity of variance by a plot of the fitted values against the residuals. If model assumptions were not fulfilled, logarithmic or square root transformations were used to stabilize variance and meet the distribution assumption.

All data were considered significant at *p* < 0.05 and at 0.05 < *p* < 0.10 as a tendency and are expressed as least square means with pooled standard error of the mean (SEM) inferred from the linear mixed effect model. The adjusted mean estimates from models with a transformed response variable are reported on the original scale after back transformation.

The levels of factors found to be significant were compared by pairwise Tukey tests using the function “glht” [44] of the R package multcomp [45].

Spearman’s rank correlations were performed to evaluate possible relationships between plasma cortisol levels and blood immune cell numbers across all time points and social ranks.

## 3. Results

### 3.1. Pregnancy-Induced Alterations in Numbers of Blood Immune Cells and Cortisol Concentration

Numbers of blood immune cells and cortisol concentrations during pregnancy are shown in Table 2. Linear-mixed effect models revealed that all physiological parameters were influenced by week of pregnancy. Post hoc analysis of pregnancy-associated alterations showed that the numbers of many lymphocytes remained constant during the first and second trimester of pregnancy (until week 7 pre partum), but decreased during the last trimester (from week 4 pre partum onwards).

Precisely, this pattern was found in T cells, NK cells, B cells, CTL, and CD8^+^ γδ-T cells (see Table 2 for statistical details). Among the TH cell subsets, a differential onset of decline was observed. The number of CD8α^+^ antigen-experienced TH cells already declined during mid-pregnancy (from week 7 pre partum) while the number of naive TH cells decreased sharply only shortly before parturition (week 2 pre partum). The change of the number of CD8^−^ γδ-T cells differed from all other subsets. Instead of declining, their number increased slightly in mid-pregnancy and returned to starting levels just at the end of pregnancy.

Analysis of antigen-presenting cell types revealed a decrease in the number of monocytes with progressing pregnancy. The number of neutrophils and, in consequence, the ratio of neutrophils:lymphocytes increased shortly before parturition. In contrast, the number of blood eosinophils decreased during pregnancy (Table 2).

Plasma cortisol concentrations increased at the end of pregnancy (week 2 pre partum), but did not differ during the beginning and middle of gestation (Table 2).

### 3.2. Effects of Social Status on Numbers of Blood Immune Cells and Cortisol Concentration

Statistical analysis further indicated that there was no significant week of pregnancy × social rank interaction. While showing differences in the absolute numbers of some important immune cells, a comparable pattern of change during pregnancy existed in sows of all three rank positions.

Table 3 indicates that the social rank influenced B cells, with MR sows showing a higher number than LR sows at week 10, 7, and 2 pre partum. Monocytes were strongly influenced by social rank, whereas neutrophils did not differ between rank positions (Table 3). Monocyte numbers of MR sows were higher than in LR sows at week 7 and 4 pre partum and also tended to be higher at week 10 pre partum. There was a tendency for a higher number of monocytes in MR sows compared to HR sows at week 4 pre partum. The number of NK cells did not differ between social rank positions of sows (Table 3).

Compared to LR sows, plasma cortisol concentrations were higher in HR sows at the beginning of pregnancy and tended to be higher in MR sows at the beginning of the second trimester. At the end of pregnancy, HR and MR sows tended to have higher plasma cortisol levels than LR sows. No differences were found between MR and HR sows (Table 3).

Although the total T cell numbers did not differ among ranks (Table 3), an in-depth analysis showed relevant differences of the T cell subsets (Table 4). At week 2 pre partum, HR sows (and MR sows with a tendency) had a lower blood number of naive TH cells than LR sows, while a higher number of blood antigen-experienced CD8α^+^ TH cells were found in HR sows. At the start of pregnancy, rank-dependent differences could be seen for γδ-T cells. At week 12 pre partum LR sows had a higher number of total γδ-T cells than MR sows which resulted from a higher number of CD8^−^ γδ-T cells. There was also a tendency for more total γδ-T cells in blood at week 10 pre partum in LR sows (Table 4).

### 3.3. Correlation between Cortisol Concentration and Blood Immune Cell Numbers

A possible relationship between cortisol levels and blood immune cell numbers was tested by Spearman’s rank correlation. Analysis across all social ranks and over all time points (*N* = 175) showed a negative correlation for the plasma cortisol concentration with the number of T cells (*r* < 0.04; ρ = −0.15), total TH cells (*r* < 0.001; ρ = −0.25), naive TH cells (*r* < 0.02; ρ = −0.18), and CD8α^+^ TH cells (*r* < 0.03; ρ = −0.17) in blood of pregnant sows.

## 4. Discussion

### 4.1. Immune Cell Numbers and Cortisol Concentration during Pregnancy

The present study provides a detailed picture of blood immune cell numbers during the entire pregnancy in sows. A decrease in the number of most lymphocyte subsets characterizes the immunological profile of sows, especially towards the end of pregnancy. This finding is generally in line with previous results in humans, rodents, and swine [1,2,4,15]. The results are also in accordance with our own previous study [16], in which blood lymphocyte subsets were analyzed in detail during the second half of gestation. This present report shows that not all lymphocyte subsets follow the same pattern, especially during the first half of pregnancy. Despite a general trend towards decreased immune cells, CD8^−^ γδ-T cells are increased at the beginning of pregnancy. This increase may be of particular relevance as γδ-T cells represent a major T cell subpopulation of peripheral blood lymphocytes in swine, which perform various important effector functions, such as cytotoxic activity or cytokine production [46]. The view that immune changes start already at early pregnancy in sows is further supported by an early decline in antigen-experienced CD8α^+^ TH cells, which play an important role in immunological memory functioning [46,47].

Two earlier studies published in 1984 and 1992 also examined total blood T-lymphocytes during the entire pregnancy in sows. The findings, however, obtained by using non-flow cytometric techniques, differ from our results and those found in other species. Georgieva [12] reported no changes for blood TH cells during pregnancy, while Schollenberger et al. [13] showed an increase of blood TH and B cells only during middle and late pregnancy.

As already demonstrated for other species [8,9,10,48], the number of blood neutrophil granulocytes increased during pregnancy in the sows examined for the present study. Since circulating granulocytes often show an activated phenotype in normal pregnancy [1,9,49], an increased number of granulocytes may partially compensate for a weakened maternal-specific immunity.

Pigs differ from other species, like rodents, insofar as the number of monocytes decreases during pregnancy. It might be possible that in pigs, increased monocytes would pose a risk for pregnancy as very high numbers of circulating monocytes correlate with preeclampsia and might cause damage to the fetus as it was shown in humans [9,50]. At present, however, the reason for this discrepancy as well as probable impacts for pregnancy in pigs is unclear and is surely a worthwhile focus for future research.

The pregnancy-associated change in peripheral lymphocyte numbers is likely to result from a redistribution of immune cells into other tissues, including the reproductive tract [51,52]. Recent studies in humans have shown that some T cell subpopulations are selectively recruited from peripheral blood to the decidua during pregnancy [52,53]. Bischof et al. [54] found pregnancy-induced changes in the uterine lymph nodes of pregnant sows with an increase in the proportion of B cells and in the CD4/CD8^H^ ratio. This redistribution might explain the decrease in numbers of blood B cells and of some T cell subsets in pregnant sows. The action of steroid hormones might be part of the underlying mechanism for this redistribution. Glucocorticoids are naturally increased at the end of pregnancy [55,56,57,58] for fetal brain and lung development, but are also major mediators of leukocyte distribution [29]. Moreover, maternal and placental hormones, such as estrogen or progesterone, could be responsible for the reduction of circulating TH and cytotoxic T cells as well as for the apoptosis of effector T cells [9,58,59,60,61].

### 4.2. Changes in Immune Cell Numbers and Cortisol Concentration Associated with Social Status

Our data show that a sow’s social status is related to blood immune cell numbers during pregnancy. Most rank-dependent particularities were seen in middle-ranking sows. In particular, the higher numbers of B cells differ from low-ranking sows and the higher number of monocytes deviates from low- and high-ranking sows. B cells actively contribute to well-being during pregnancy, for example, by the production of protective antibodies. They may, however, also contribute to pregnancy-associated pathologies because of the production of autoantibodies [62]. A higher number of B cells and monocytes in middle-ranking sows may therefore point to imbalances between immune activation and tolerance. Moreover, an effective immune response requires an appropriate migration pattern of B cells to lymph nodes or other relevant lymphatic organs [63,64]. Higher numbers of B cells in middle-ranking sows may indicate that this process is impaired. An elevated number of B cells may also stem from an extended life span of B cells. In middle-ranking sows, this would mean a deviation from the “normal” status in which the majority of the mature B lymphocytes in the periphery have a short life expectancy [65].

Why is it justified to assume that middle-ranking sows deviate from normal status and why do these sows differ from low- and high-ranking sows? Middle-ranking sows have to challenge dominant sows to improve their social status while simultaneously defending their own status against subdominants. According to Mendl et al. [27], high social status (and continued success) as well as the acceptance of low status (and no success) have lower physiological costs than a middle rank status. Under such conditions, increased cortisol levels were found in middle-ranking sows [26,27]. Although in the present study, blood cortisol concentrations were just slightly increased in middle-ranking sows, the immunological parameters point to stressful social conflicts during dominance establishment [66,67]. Monocytosis [17], lower numbers of some T cell populations compared to HR sows at some points, and a negative correlation between cortisol levels and numbers of blood T and TH cells support the assumption of a contribution of the hypothalamus-pituitary-adrenal (HPA) axis hormones to the immune cells of pregnant sows. The effect on B cells in MR sows could also be explained by glucocorticoid action, because stressful conditions or treatment with dexamethasone have been shown to increase the number of B cells [63,68,69]. However, it should be noted that the stress effects on B cells are complex, as decreased B cell numbers have also been observed in response to stressors [67,70]. It is apparent that more research is needed to clarify the interactions of social stress and rank position and their effects on immune cell distribution to unravel these associations.

Future studies should investigate the hormonal influences in more detail. It is known that plasma levels of cortisol can be influenced by the time of sampling, as the activity of the HPA axis is highly variable and porcine plasma cortisol concentrations were also found to show their highest peak in the morning [36,71]. Cortisol levels thus might have been elevated before sampling in the middle-ranking sows of the present study, but had already declined to baseline at the time of measurement. More insight will also come from assessment of corticosteroid-binding globulin as well as catecholamines. Moreover, although stress is a prime candidate for explaining the immune status in middle-ranking sows, it might not be the only factor. Rank-dependent differences in physical activity or a shift in diurnal rhythms may also play a role [36].

Finally, it should be recognized that social rank, although associated with altered absolute blood immune cell numbers, did not disrupt the normal pattern of pregnancy-associated immune changes in sows. This would suggest that a finely tuned balance between innate and adaptive immune cells is of essential importance for a successful pregnancy and is relatively resistant against stress-induced alterations.

## 5. Conclusions

The analysis of blood immune cell subsets in sows shows that pregnancy-associated immunomodulations exist in each trimester of pregnancy and future studies on the effects of social stress on the immune system in pregnant pigs should ideally cover the entire gestation period. The rank-specific analysis revealed that middle-ranking sows showed distinct changes in the immune system, such as monocytosis and a decrease of some T cell subsets. These immunological changes are best interpreted as a stress response, but some open questions remain, such as the role of cortisol and the effects on B cells. The results of the present study are of relevance for livestock production as they indicate that a low social status may not (always) be the most stressful condition. Instead, it appears that middle-ranking positions in group-housing environments could be associated with more adverse effects on welfare and health.

## Figures and Tables

**Table 1:** Ethogram of aggressive and submissive behavior observed in pregnant sows.

Behavior	Description
**Aggressive**	
Biting	Biting with teeth at another sow’s head and body. Mouth of the acting sow is open. The attempt is also evaluated.
Head-to-body/head knocking	A rapid, heavy thrust or push upwards or sideways with head or snout against another sow’s body or head.
Parallel pressing	Two sows standing side by side and pushing their shoulders and bodies against each other. With or without biting and head-to-head knock.
Inverse parallel pressing	Two sows standing face front to front and push their shoulders, bodies and heads against each other. With or without biting and head-to-body knock.
Following/Chasing	Moving at a walking or running pace more than 3 steps in pursuit of another sow and reducing the distance between both animals to less than 1 m. The receiver sow withdraws or flees.
Displacing	Forcing another sow to leave and avoid its current location, lying place, trough, or drinker by appearance alone, without any physical contact. The receiver sow avoids the intruder.
**Submissive**	
Avoiding	Result of “displacing”. Leaving and avoiding (>2 steps) the current location, lying place, trough, or drinker caused only by another sow’s appearance, not by any physical contact.
Withdrawing	Possible result of any aggressive behavior. Moving away (>2 steps) from another sow at a walking pace.
Fleeing	Possible result of any aggressive behavior. Moving away (>3 steps) from another sow at a running pace.

**Table 2:** Count of blood leukocytes and lymphocyte subpopulations in sows at defined weeks pre partum.

Count/µL Blood	Weeks Pre Partum						*p*-Value
	12	10	7	4	2	Pooled SEM	Week of Pregnancy
Lymphocytes (L) ^†^	5488 ^a^	5349 ^a^	5298 ^a^	4882 ^b^	4170 ^c^	356	<0.001
T cells ^‡^	4537 ^a^	4458 ^a^	4316 ^ab^	4100 ^b^	3383 ^c^	162	<0.001
B cells ^†^	840 ^ab^	774 ^a^	860 ^b^	683 ^c^	689 ^c^	167	<0.001
NK cells ^†^	121 ^a^	112 ^a^	120 ^a^	108 ^a^	86 ^b^	20	<0.001
Cytotoxic T cells ^‡^	1162 ^a^	1099 ^ab^	1111 ^a^	1011 ^b^	847 ^c^	100	<0.001
Total TH cells ^†^	2359 ^a^	2291 ^ab^	2101 ^b^	2121 ^b^	1699 ^c^	91	<0.001
Naive TH cells ^†^	549 ^a^	573 ^a^	518 ^a^	537 ^a^	433 ^b^	56	<0.001
CD8α^+^ TH cells ^†^	1736 ^a^	1647 ^ab^	1523 ^b^	1518 ^b^	1225 ^c^	72	<0.001
Total γδ-T cells	1002 ^ab^	1046 ^a^	1032 ^a^	913 ^b^	805 ^c^	102	<0.001
CD8^+^ γδ-T cells ^‡^	751 ^a^	759 ^a^	745 ^a^	645 ^b^	563 ^c^	78	<0.001
CD8^−^ γδ-T cells ^‡^	202 ^a^	233 ^b^	242 ^b^	224 ^ab^	198 ^a^	20	<0.001
Neutrophils (N) ^†^	3101 ^a^	2985 ^a^	2908 ^a^	2947 ^a^	3552 ^b^	143	<0.001
Eosinophils ^‡^	532 ^a^	457 ^bc^	428 ^bc^	483 ^ab^	412 ^c^	34	<0.001
Monocytes ^†^	668 ^a^	641 ^ab^	663 ^ab^	614 ^ab^	602 ^b^	50	<0.05
Ratio of N:L ^†^	0.6 ^a^	0.6 ^a^	0.6 ^a^	0.6 ^a^	0.9 ^b^	0.05	<0.001
Cortisol ^†^, ng/mL plasma	16.4 ^a^	16.9 ^a^	18.9 ^a^	19.0 ^a^	25.0 ^b^	1.92	<0.001

Data are expressed as least-square means with pooled standard error of the mean (SEM). Adjusted mean estimates from models with ^†^ logarithmic or ^‡^ square root transformed response variable are reported on the original scale after back transformation. *p*-Values indicate a significant effect of the week of pregnancy. Different superscripts ^a, b, c^ within a row indicate the weeks of pregnancy (12 vs. 10, 12 vs. 7, 12 vs. 4, 12 vs. 2, 10 vs. 7, 10 vs. 4, 10 vs. 2, 7 vs. 4, 7 vs. 2, 4 vs. 2) that significantly differ after post-hoc testing (*p* < 0.05) for the respective immune cell type.

**Table 3:** Influence of social rank on count of blood immune cells and plasma cortisol concentration in high-ranking (HR), middle-ranking (MR), and low-ranking (LR) pregnant sows at defined weeks pre partum.

Item (Count/µL)	Rank Position	12	Pooled SEM	*p*-Value	10	Pooled SEM	*p*-Value	7	Pooled SEM	*p*-Value	4	Pooled SEM	*p*-Value	2	Pooled SEM	*p*-Value
Lymphocytes	HR	^†^ 5048	533	0.2	5831	440	0.7	^†^ 5634	410	0.64	^†^ 5199	512	0.67	^†^ 4482	386	0.49
	MR	^†^ 5827			5541			^†^ 5349			^†^ 4802			^†^ 4044		
	LR	^†^ 5846			5490			^†^ 5045			^†^ 4764			^†^ 4112		
T cells	HR	^†^ 4701	355	0.13	4701	340	0.77	^†^ 4444	353	0.72	4385	331	0.74	3576	267	0.45
	MR	^†^ 4090			4361			^†^ 4152			4067			3240		
	LR	^†^ 4837			4531			^†^ 4300			4100			3515		
B cells	HR	^†^ 977	153	0.47	936 ^ab^	163	0.05	887 ^ab^	131	0.02	751	160	0.12	746 ^ab^	127	0.04
	MR	^†^ 843			919 ^a^			1108 ^a^			793			821 ^a^		
	LR	^†^ 769			670 ^b^			730 ^b^			624			619 ^b^		
NK cells	HR	^†^ 159	34	0.08	^†^ 144	30	0.23	^†^ 152	19	0.31	^†^ 123	26	0.4	^†^ 99	30	0.24
	MR	^†^ 101			^†^ 100			^†^ 114			^†^ 96			^†^ 73		
	LR	^†^ 139			^†^ 110			^†^ 106			^†^ 112			^†^ 93		
Neutrophils	HR	3108	294	0.74	2916	328	0.81	3001	244	0.98	^†^ 2499	245	0.14	^†^ 2957	333	0.2
	MR	3353			3277			2960			^†^ 3096			^†^ 3690		
	LR	3110			3140			2950			^†^ 3078			^†^ 3781		
Monocytes	HR	^†^ 689	53	0.38	^†^ 593 ^ab^	45	0.08	759 ^ab^	46	0.02	604 ^at^	55	0.003	589	67	0.26
	MR	^†^ 703			^†^ 705 ^a^			745 ^a^			728 ^b^			656		
	LR	^†^ 623			^†^ 593 ^bt^			570 ^b^			534 ^a^			585		
Cortisol, ng/mL plasma	HR	^†^ 31.8 ^a^	2.9	0.02	20.7 ^ab^	3.3	0.09	22.5	3.1	0.13	24.4	2.9	0.26	32.7 ^a^	2.3	0.06
	MR	^†^ 26.0 ^ab^			22.0 ^at^			23.1			18.4			27.4 ^a^		
	LR	^†^ 20.0 ^b^			14.6 ^b^			17.5			21.0			20.4 ^bt^		

Data are expressed as least-square means with pooled standard error of the mean (SEM). Adjusted mean estimates from models with ^†^ logarithmic transformed response variable are reported on the original scale after back transformation. *p*-Values indicate a significant effect of social rank at the respective week of pregnancy. Different superscripts ^a, b^ within a column indicate the social rank positions (HR vs. MR, HR vs. LR, MR vs. LR) that significantly differ after post-hoc testing (*p* < 0.05) at the respective week pre partum for each immune cell type. Different superscripts ^at, bt^ within a column indicate the social rank positions (HR vs. MR, HR vs. LR, MR vs. LR) that tended to differ (*p* < 0.01) for each immune cell type at the respective week pre partum (HR with *N* = 8; MR with *N* = 15; LR with *N* = 12).

**Table 4:** Influence of social rank on the count of blood T cell subpopulations in high-ranking (HR), middle-ranking (MR), and low-ranking (LR) pregnant sows at defined weeks pre partum.

Item (Count/µL)	Rank Position	12	Pooled SEM	*p*-Value	10	Pooled SEM	*p*-Value	7	Pooled SEM	*p*-Value	4	Pooled SEM	*p*-Value	2	Pooled SEM	*p*-Value
Total TH cells	HR	^†^ 2374	194	0.52	2500	237	0.69	^†^ 2078	181	0.99	^†^ 2377	225	0.28	^†^ 1854	144	0.47
	MR	^†^ 2215			2299			^†^ 2062			^†^ 2074			^†^ 1637		
	LR	^†^ 2477			2286			^†^ 2090			^†^ 2050			^†^ 1722		
Naive TH cells	HR	^†^ 420	80	0.14	^†^ 495	120	0.59	^†^ 406	70	0.23	508	121	0.74	^†^ 303 ^a^	51	0.02
	MR	^†^ 568			^†^ 599			^†^ 550			585			^†^ 429 ^bt^		
	LR	^†^ 682			^†^ 620			^†^ 578			645			^†^ 508 ^b^		
CD8α^+^ TH cells	HR	^†^ 1859	158	0.60	^†^ 1797	161	0.33	^†^ 1694	153	0.76	^†^ 1867	175	0.21	1609 ^a^	119	0.04
	MR	^†^ 1642			^†^ 1650			^†^ 1486			^†^ 1504			1201 ^b^		
	LR	^†^ 1681			^†^ 1488			^†^ 1403			^†^ 1392			1246 ^bt^		
CTL	HR	^†^ 1161	163	0.22	1090	147	0.98	1092	123	0.95	1072	146	0.85	966	94	0.32
	MR	^†^ 1023			1122			1132			1004			812		
	LR	^†^ 1245			1125			1137			1057			879		
Total γδ-T cells	HR	1094 ^at^	176	0.006	1146 ^ab^	152	0.07	1020	102	0.75	905	182	0.38	750	189	0.15
	MR	803 ^b^			890 ^a^			1005			828			707		
	LR	1236 ^a^			1172 ^bt^			1092			972			885		
CD8^+^ γδ-T cells	HR	793	158	0.64	845	106	0.07	774	130	0.88	689	130	0.34	^†^ 555	118	0.70
	MR	655			683 ^a^			744			608			^†^ 514		
	LR	917			837 ^bt^			785			689			^†^ 561		
CD8^−^ γδ-T cells	HR	^†^150 ^at^	32	0.02	^†^ 178	38	0.26	257	52	0.68	224	57	0.34	166	67	0.11
	MR	^†^160 ^a^			^†^ 194			259			210			177		
	LR	^†^266 ^b^			^†^ 248			303			288			276		

Data are expressed as least-square means with pooled standard error of the mean (SEM). Adjusted mean estimates from models with ^†^ logarithmic transformed response variable are reported on the original scale after back transformation. *p*-Values indicate a significant effect of social rank at the respective week of pregnancy. Different superscripts ^a, b^ within a column indicate the social rank positions (HR vs. MR, HR vs. LR, MR vs. LR) that significantly differ after post-hoc testing (*p* < 0.05) at the respective week pre partum for each immune cell type. Different superscripts ^at, bt^ within a column indicate the social rank positions (HR vs. MR, HR vs. LR, MR vs. LR) that tended to differ (*p* < 0.01) for each immune cell type at the respective week pre partum (HR with *N* = 8; MR with *N* = 15; LR with *N* = 12).
